# Temporal changes and correlates of tobacco and E-cigarettes use among school-going students in Albania: insights from global youth tobacco surveys (2015–2020)

**DOI:** 10.1007/s00787-024-02629-x

**Published:** 2024-12-21

**Authors:** Omid Dadras

**Affiliations:** 1 Department of Global Public Health and Primary Care, University of Bergen, Årstadveien 17, Bergen, 5009 Norway; 2 Research Center for Child Psychiatry, University of Turku, Turku, Finland

**Keywords:** Tobacco use, E-cigarettes, Smoking, adolescents, Temporal changes, Correlates, Albania

## Abstract

Tobacco use often starts during adolescence, with many adults beginning before 21. This study investigated the patterns and factors associated with tobacco and e-cigarette use among school-aged adolescents in Albania. Data were analyzed from two Albania Global Youth Tobacco Surveys (GYTS) conducted in 2015 and 2020. Participants included 7th-10th grade students (ages 12–16) who completed the survey (*n* = 9985). Descriptive statistics depicted demographic characteristics, tobacco, and e-cigarette use across survey years. Logistic regression assessed temporal changes and the odds of use across independent variables, adjusting for age and sex. From 2015 to 2020, tobacco and e-cigarette use among students aged 13–15 increased from 12.86 to 14.49% and from 6.8 to 8.8%, respectively. Cigarette smoking declined from 8.4 to 4.3%, but the use of other tobacco products nearly doubled, from 6.4 to 12.3%. Higher odds of use were found among males, older students, and those with more pocket money. Environmental factors like family, peer, and media exposure to smoking increased the likelihood of use. Anti-tobacco messages had no significant impact, while advertising strongly influenced usage. Knowledge of tobacco harms and anti-smoking attitudes correlated with lower odds of use. Increased availability of cigarettes near schools was linked to higher odds of tobacco use. The study highlights gaps in anti-tobacco messaging and the strong influence of advertising, underscoring the need for targeted and effective tobacco control strategies in Albania.

## Introduction

Tobacco use is a leading cause of preventable death and disease worldwide, with more than eight million annual deaths attributed to tobacco use, including 1.3 million non-smokers exposed to second-hand smoke [1]. Tobacco use often starts during adolescence, with most adults who currently smoke tobacco having initiated before age 21 [2]. While there has been a global decline in cigarette smoking among adults, the use of e-cigarettes (a typical nicotine delivery system that does not contain tobacco) and other tobacco products has increased, particularly among young adults [3]. Among students in US middle and high schools, e-cigarettes are now the most commonly used tobacco product, with a higher prevalence among certain subpopulations [4, 5]. Adolescents who use tobacco are at increased risk for immediate and long-term health issues, including impaired lung development, respiratory illnesses, and nicotine addiction [6]. This can affect brain development, impair cognitive functions, and elevate the risk of mood disorders like anxiety and depression [7]. Additionally, emerging research links affective temperaments, especially hopelessness and anxiety, with tobacco use [8, 9]. Furthermore, adolescence is a time of heightened vulnerability to psychosocial stressors, including those exacerbated by the COVID-19 pandemic after 2019 [10]. These factors, in addition to the mental health challenges adolescents face, may heighten their risk for tobacco dependence and psychopathology. Therefore, addressing tobacco use, including emerging products, among youth is crucial to prevent a new generation from becoming addicted and suffering long-term health consequences.

Tobacco use is a significant public health issue in Albania, with high smoking prevalence among both men and women, particularly in urban areas [11]. According to the World Health Organization (WHO) data reported in 2020, an estimated 23.4% of Albanian adults including 39.8% of men and 6.6% of women currently smoke tobacco [12]. This is higher than some neighboring Balkan countries such as North Macedonia (22.6%), Montenegro (21.8%), and Bosnia & Herzegovina; however, lower than Greece (35%) and Bulgaria (27.3%) [13]. Among students aged 13–15 years, the current use of tobacco products is 15.3%, with a higher prevalence in boys (20.2%) than girls (9.9%). Approximately 4.4% of students, 6.4% of boys, and 2.4% of girls currently smoke cigarettes, according to 2020 data [14]. While the overall adult smoking rate in Albania has gradually declined over the past two decades, it remains high, especially among men, with an estimated prevalence of 40–51% as of the latest data [12, 15]. The decline has been more pronounced among women and youth. However, smoking rates are still concerningly high, particularly for younger adolescent boys, indicating more efforts are needed to reduce tobacco use prevalence in Albania [16].

Tobacco use imposes a significant economic burden on Albania, with the cost of smoking estimated at $150 million USD annually, including direct healthcare costs and lost productivity. Cigarettes, priced at approximately 250 ALL ($2.37 USD) per pack, represent a significant financial burden, particularly for lower-income families. Smokers spend 4.56% of GDP per capita annually on tobacco, diverting resources from essential needs [17]. Albania has a ban on smoking in most public places, including healthcare facilities, educational facilities, government facilities, indoor offices, restaurants, pubs and bars. A law adopted in 2006 prohibits all types of advertising, promotion, and sponsorship of tobacco on radio, TV, and print media; and establishes a minimum age for tobacco sale [18]. The legislation was amended in 2013 (monitor informal tobacco production, sales and advertising and increasing the maximum fines for violation from the law) [18]. However, compliance with the smoke-free laws appears to be low, scoring only 3 out of 10 in the WHO report on the global tobacco epidemic [19].

The period from 2015 to 2020 marks a critical period during which traditional forms of tobacco consumption have intersected with the rising prevalence of e-cigarettes, particularly among adolescents and young adults [20]. Albania amended the tobacco ban law in 2019 (in law number 56/2019 article 4- point 9 and article 5-point 10, the words electronic cigarettes were added) to regulate the use of e-cigarettes [21]; however, a study published in 2021 suggested a rising trend in e-cigarette use among youth in 17 WHO European Region countries in including Albania, with many more males than females reporting the use of e-cigarettes [22]. However, this study did not specify the temporal changes and specific correlates of e-cigarettes among Albanian adolescents. Evidence suggests e-cigarettes are contributing to increased nicotine exposure and are associated with initiating combustible cigarette use among adolescents [23–25]. This raises urgent questions about trends, correlates, and broader implications for public health and policy in Albania, particularly regarding taxation, law enforcement, cessation services, and youth-focused public education campaigns.

Against this background, the study aims to provide insights into temporal changes in tobacco use and e-cigarettes and identify the correlates that drive these behaviors among school-going adolescents in Albania. The findings can inform and guide public health policies and interventions to prevent and reduce tobacco use and e-cigarettes among adolescents in Albania. It can also contribute to the global understanding of the prevalence and trends of tobacco use and e-cigarettes among school-going students, which is essential for developing effective tobacco control policies and interventions.

## Materials and methods

### Data sources

We used the data from two consecutive Albania Global Youth Tobacco Surveys (GYTS) conducted in 2015 and 2020. GYTS is a cross-sectional, nationally representative school-based survey of students in grades 7-10th, associated with ages 13 to 15 years. It uses a standard core questionnaire, sample design, and data collection protocol to generate comparable data within and across countries. GYTS is an important and dynamic monitoring procedure to reach the goals of the World Health Organization (WHO) Framework Convention on Tobacco Control. The 2015 and 2020 Albania GYTSs were funded and received technical support from the WHO and the US Centers for Disease Control and Prevention (CDC).

### Survey design, sampling, and participants

GYTS uses a two-stage sampling design, selecting schools with probability proportional to enrollment size, followed by random selection of classes within the schools. All students in the selected classes are eligible to participate. The 2015 survey, conducted by the Ministry of Health and the Institute for Nature Conservation in Albania (INCA), had a response rate of 89.3%, with 4,672 students completing the survey (3,482 aged 13–15). The 2020 survey, conducted by the University of Medicine, Tirana, in collaboration with the Ministry of Health and Social Protection, had a 90.4% response rate, with 5,388 students completing the survey (4,052 aged 13–15). High response rates in both surveys indicate strong engagement and ensure the data represent the target population. This study includes all participants in grades 7–10 (*n* = 9,985, 98% aged 12–16).

### Outcome variables

*Tobacco Use* refers to the consumption of any product containing tobacco, as measured within a specific timeframe (e.g., past 30 days). It encompasses three main categories:

- *Cigarette Smoking*: This includes the inhalation of burning tobacco from roll-your-own cigarettes, commercially manufactured cigarettes, or any product delivering nicotine through the combustion of tobacco (e.g., kreteks, bidis).
- Smokeless Tobacco: This category encompasses any non-combustible tobacco product typically placed in or against the oral cavity for prolonged periods. Examples include chewing tobacco (loose leaf, plugs), snuff (powdered tobacco), and dissolvable tobacco products.
- Other Tobacco Products: This category includes various tobacco products besides cigarettes and smokeless tobacco. This could include cigars, mini-cigars, cigarillos, pipes, waterpipes (shisha/hookah), and bidis (depending on the specific classification system used).

*E-cigarette use* was as using electronic cigarettes in the past 30 days.

### Independents variables

The choice of independent variables linked to tobacco use and e-cigarettes was guided by previous relevant studies in the adolescent population [26–28]. The variables were classified into six categories as follows:

#### Sociodemographic factors

Age groups (< 14, 14–15, > 15); sex; grade (7-10th); pocket money (measured by asking “During an average week, how much money do you have that you can spend on yourself, however you want?” responses were coded into three categories (terciles) including 1 = < 100, 2 = 100–500, 3 = > 500 Albanian Lek).

#### Exposure to smoking

parental/sibling/close friend/classmate smoking, ever witnessed a teacher smoking in school (yes, no), witnessed someone smoking inside home/ enclosed public places or at outdoor public places in the past 7 days (yes, no), witnessed someone smoking at school or on TV/in movies in the last 30 days (yes, no).

#### Anti-tobacco messages

Have seen or heard anti-tobacco messages on social media or at social events in last 30 days (yes, no), have seen signs forbidding buying tobacco in last 30 days (yes, no).

#### Advertisements/promotions

Have seen videos on the internet encouraging smoking in last 30 days (yes, no), have seen tobacco ads at social events/sales points in last 30 days (yes, no), ever offered free tobacco products from a company (yes, no).

#### Knowledge/attitude toward smoking

Awareness of tobacco smoke harm (yes, no), taught about tobacco harms in school during last year (yes, no), cigarette smoking is joyful (yes, no), willing to use a cigarette if offered by a friend (yes, no), it is difficult to quit smoking once started (yes, no), support for smoking bans in indoor public places (yes, no), support tobacco advertising ban (yes, no).

#### Availability of tobacco

Availability of cigarettes near school (yes, no), refused to be sold cigarettes due to age in last 30 days (yes, no), difficulty in buying cigarettes (easy, difficult).

### Statistical analysis

The datasets from GYTS 2015 and 2020 were separately analyzed to identify, compare, and contrast the factors associated with the use of tobacco and e-cigarettes between two surveys and then used pool analysis by combing the two datasets. Descriptive statistics were employed to describe the distribution of demographic characteristics, the prevalence of tobacco use and its sub-categories as well as e-cigarette use among students in grades 7-10th across different survey years and independent variables. The results were reported as frequency (%) for descriptive analysis. The chi-square test was used to examine the relationship between each independent variable and the use of tobacco or e-cigarettes in each survey. The temporal changes in the use of tobacco and e-cigarettes were examined by logistic regression analysis including the survey year as the independent variable and adjusting for the difference in age and sex distribution between two surveys. In addition, the odds of tobacco use and e-cigarettes across selected independent variables were examined by logistic regression adjusting for age and sex. The aim was to identify factors associated with tobacco and e-cigarette use, rather than create a predictive model. By focusing on independent effects and controlling for age and sex, we can better understand how various factors influence these behaviors and assess the individual impact of each study variable on tobacco and e-cigarette use [29]. The results of regression analysis were reported as odds ratios (ORs) and 95% confidence intervals (CIs). Due to the multi-stage design of GYTS and accounting for non-response, sampling weights were calculated and applied in data analysis. The analysis was performed using STATA 17 and *p* < 0.05 was set as the statistical significance margin.

## Results

### Sociodemographic characteristics, by survey year

Except for age distribution, there were no significant differences in the demographic characteristics of participants between GYTS 2015 and 2020 (Table 1).

**Table 1:** Distribution of demographic characteristics of Albanian students in grades 7-10th, by survey year

	Survey year		
	2015	2020	
	N^1^ (weighted%)	N^1^ (weighted%)	p-value
**Age (years)**			
< 14	1307 (25.6)	2446 (44.7)	
14–16	2391 (52.0)	2605 (49.1)	
> 16	928 (22.3)	308 (6.1)	< 0.001
**Sex**			
Female	2224 (53.1)	2587 (50.9)	
Male	2396 (46.9)	2739 (49.2)	0.276
**Grade**			
7th	1170 (22.2)	1292 (24.0)	
8th	1159 (26.2)	1380 (25.1)	
9th	1222 (25.9)	1348 (25.4)	
10th	1083 (25.7)	1313 (25.5)	0.867
**Total**	4626 (100)	5359 (100)	

^1^ The sum may not add up to the total number due to missing data

### Changes and patterns of tobacco use and e-cigarettes

The overall tobacco use increased from 12.9% in 2015 to 14.5% in 2020 among Albanian students in grades 7-10th and the odds of tobacco use was 1.57 (95% CI: 1.26–1.94) higher among students in 2020 as compared to 2015. Similarly, the use of e-cigarettes increased from 6.8% in 2015 to 8.8% in 2020; with substantially higher odds in 2020 (OR: 1.64; 95% CI: 1.30–2.08). Exploring the tobacco use patterns, there was a significant decline in cigarette smoking from 8.4% in 2015 to 4.3% in 2020 with 30% lower odds of cigarette smoking in 2020 (OR: 0.70, 95% CI: 0.54–0.91). On the contrary, the consumption of other tobacco products significantly increased from 6.4% in 2015 to 12.3% in 2020 with approximately three times higher odds in 2020 (OR: 2.87, 95% CI: 2.29–3.62). No significant trend was observed for smokeless tobacco. The dual use increased from 3.3 to 4.8% with 1.98 times higher odds in 2020 as compared to 2015.

**Table 2:** Prevalence and changes in tobacco use and e-cigarettes between 2015 to 2020 among Albanian students in grades 7-10th

	Survey year		
	2015	2020	
	N (weighted%)	N (weighted%)	OR (95% CI)^1^
**Tobacco use**	610 (12.9)	765 (14.5)	1.57 (1.26–1.94)*
Cigarette	370 (8.4)	224 (4.3)	0.70 (0.54–0.91)*
Other tobacco products	300 (6.4)	583(12.3)	2.87 (2.29–3.62)*
Smokeless tobacco	110 (2.3)	135 (2.6)	1.20 (0.83–1.75)
**E-cigarettes**	317 (6.8)	462 (8.8)	1.64 (1.30–2.08)*
**Dual use**	158 (3.3)	255 (4.8)	1.98 (1.43–2.74)*

^1^ Adjusted for age and sex

### Sociodemographic correlates of use of tobacco or e-cigarettes

There was a notable association between age and the use of both tobacco and e-cigarettes as depicted in Table 3. Students aged 14–15 years showed increased odds of using tobacco (OR: 1.95) and e-cigarettes (OR: 1.65) compared to those under 14. This likelihood further escalated in students over 15 years for tobacco (OR: 3.96) and e-cigarettes (OR: 2.88). The use and likelihood of tobacco and e-cigarettes were significantly lower among females in both surveys and pooled datasets. In terms of grade levels, students in grades 8-10th were more likely to use tobacco, particularly those in 9th and 10th grades, who also showed higher odds of using e-cigarettes. Specifically, 10th graders had the highest odds of tobacco use (OR: 3.60, 95% CI: 2.78–4.66) and e-cigarette use (OR: 2.31, 95% CI: 1.69–3.16). Having more than 500 ALL pocket money was associated with higher odds of using both tobacco (OR: 2.48) and e-cigarettes (OR:2.27).

### Exposure to smoking and use of tobacco or e-cigarettes

As Table 4 illustrates, the odds of using both tobacco (OR:1.34) and e-cigarettes (OR:1.21) was higher for students with at least one parent smoking. The presence of a smoking sibling dramatically increased the odds of both tobacco (OR: 2.90) and e-cigarettes (OR: 2.73) use. Students with smoking friends were significantly more likely to use tobacco (OR: 3.78) and e-cigarettes (OR: 3.13), underlining the impact of peer smoking. The odds of using tobacco and e-cigarettes were higher for students with smoking classmates, with ORs ranging from 2.31 to 4.99, depending on the proportion of smoking classmates. Witnessing a teacher smoking in school was associated with higher odds of both tobacco (OR: 2.27) and e-cigarette use (OR: 2.23). Students who witnessed smoking at home or in enclosed public places had increased odds of tobacco and e-cigarette use, with ORs above 2.0 in all cases. Exposure to smoking on TV or in movies was also linked to higher odds of tobacco (OR: 1.62) and e-cigarette use (OR: 1.55).

### Anti-tobacco messages and use of tobacco or e-cigarettes

There was no significant association between exposure to anti-tobacco messages on social media or at social events and the use of tobacco or e-cigarettes among adolescents in this study, as shown in Table 5. For example, adolescents who reported seeing anti-tobacco messages on social media in the past 30 days had slightly lower odds of using tobacco and e-cigarettes compared to those who did not; however, these differences were not statistically significant (OR: 0.88, 95% CI: 0.77–1.00 for tobacco; OR: 0.87, 95% CI: 0.71–1.07 for e-cigarettes). Similarly, seeing signs forbidding the purchase of tobacco in the last 30 days and exposure to anti-tobacco messages at social events showed no significant impact on tobacco or e-cigarette use.

### Advertisements/promotions and use of tobacco or e-cigarettes

As Table 6 indicates, students who reported seeing internet videos promoting tobacco smoking in the last 30 days showed significantly higher odds of using tobacco (OR: 1.94) and e-cigarettes (OR: 2.32) compared to those who did not see such videos. Exposure to tobacco ads and promotions at social events was also associated with higher odds of tobacco use (OR: 1.89) and e-cigarette use (OR: 2.30). Seeing cigarette ads in sales points correlated with a higher likelihood of tobacco use (OR: 1.75) and e-cigarettes (OR: 1.81). Students ever offered free tobacco products by tobacco companies had substantially higher odds of tobacco use (OR: 3.16) and e-cigarette use (OR: 2.52).

### Knowledge/attitude toward smoking and use of tobacco or e-cigarettes

The associations between knowledge and attitude toward smoking and the use of tobacco or e-cigarettes are described in Table 7. A significant reduction in tobacco and e-cigarette use was observed among students aware of the harms of tobacco smoke, with the odds ratio (OR) being 0.39 for both tobacco and e-cigarette use compared to those unaware. Students taught about tobacco harms in school last year showed a lower likelihood of using tobacco (OR: 0.76) and e-cigarettes (OR: 0.78) compared to those not taught. Students who believe cigarette smoking is joyful had markedly higher odds of using tobacco (OR: 4.75) and e-cigarettes (OR: 3.96). A strong correlation was found between the willingness to use a cigarette if offered by a friend and actual use, with ORs of 6.91 for tobacco and 4.26 for e-cigarettes. Those who believe it is difficult to quit smoking were less likely to use tobacco (OR: 0.64) and e-cigarettes (OR: 0.61). The likelihood of tobacco and e-cigarettes was 50% lower among students supporting bans in indoor public places. There was a lower likelihood of tobacco (OR: 0.65) and e-cigarette (OR: 0.71) use among students who support a tobacco advertising ban.

### Availability of tobacco and use of tobacco

The odds of tobacco use were 2.5 times higher among students who reported the availability of cigarettes near their school. No significant relationship was observed between the refusal to sell cigarettes to students due to their age and tobacco use, likewise for difficulty in buying cigarettes (Table 8).

**Table 3:** Sociodemographic factors and use of tobacco or e-cigarettes among Albanian students in grades 7-10th, by survey year

	Tobacco use			E-cigarettes		
	Survey year		Pooled dataset	Survey year		Pooled dataset
	2015	2020		2015	2020	
	N (%)	N (%)	OR (95% CI)	N (%)	N (%)	OR (95% CI)
**Age (years)**						
< 14	72 (5.3)	245 (10.3)	Reference	64 (4.3)	146 (6.2)	Reference
14–15	302 (11.7)	434 (17.0)	1.95 (1.60–2.38)*	152 (6.4)	261 (10.3)	1.65 (1.34–2.05)*
> 15	211 (22.3)	77 (23.7)	3.96 (3.10–5.07)*	101 (10.8)	51 (16.1)	2.88 (2.18–3.80)*
*p-value*	< 0.001	< 0.001		< 0.001	< 0.001	
**Sex**						
Female	389 (16.7)	504 (19.5)	0.40 (0.35–0.47)*	230 (9.9)	348 (13.4)	0.28 (0.23–0.35)*
Male	194 (7.5)	241 (8.9)	Reference	84 (3.2)	105 (3.9)	Reference
*p-value*	< 0.001	< 0.001		< 0.001	< 0.001	
**Grade**						
7th	61 (4.8)	110 (8.8)	Reference	58 (4.4)	73 (5.8)	Reference
8th	115 (8.5)	173 (13.0)	1.66 (1.29–2.14)*	67 (5.0)	107 (8.1)	1.29 (0.97–1.70)
9th	181 (14.6)	226 (17.6)	2.70 (2.10–3.48)*	80 (6.8)	127 (9.6)	1.24 (1.24–2.24)*
10th	236 (21.4)	248 (18.3)	3.60 (2.78–4.66)*	106 (10.4)	148 (11.4)	2.31 (1.69–3.16)*
*p-value*	< 0.001	< 0.001		< 0.001	< 0.001	
**Pocket money (ALL)** ^**1**^						
< 100	80 (8.2)	139 (9.2)	Reference	45 (3.7)	86 (5.98)	Reference
100–500	193 (8.8)	287 (11.5)	1.12 (0.95–1.33)	117 (5.6)	166 (6.7)	1.25 (0.96–1.63)
> 500	311 (21.2)	322 (26.1)	2.48 (2.11–2.92)*	152 (10.9)	197 (15.9)	2.27 (1.73–2.97)*
*p-value*	< 0.001	< 0.001		< 0.001	< 0.001	

* p-value < 0.05. ^1^ Adjusted for age and sex

**Table 4:** Exposure to smoking and use of tobacco or e-cigarettes among Albanian students in grades 7-10th, by survey year

	Tobacco use			E-cigarettes		
	Survey year		Pooled dataset	Survey year		Pooled dataset
	2015	2020		2015	2020	
	N (%)	N (%)	OR (95% CI)^1^	N (%)	N (%)	OR (95% CI)^1^
**Parental smoking**						
No	332 (11.3)	382 (12.6)	Reference	182 (6.5)	226 (7.5)	Reference
Yes	243 (14.1)	348(16.6)	1.34 (1.15–1.55)*	125 (6.9)	205 (9.9)	1.21 (1.01–1.44)*
*p-value*	0.029	0.003		0.635	0.007	
**Sibling smoking**						
No	275 (9.5)	305 (10.8)	Reference	134 (5.1)	204 (7.2)	Reference
Yes	113 (23.3)	126 (27.5)	2.90 (2.28–3.68)*	63 (11.8)	87 (19.4)	2.73 (2.07–3.59)*
*p-value*	< 0.001	< 0.001		< 0.001	< 0.001	
**Close friend smoking**						
No	153 (5.5)	402 (10.0)	Reference	97 (3.6)	237 (6.0)	Reference
Yes	431 (23.4)	344 (29.8)	3.78 (3.30–4.33)*	216 (11.9)	215 (18.8)	3.13 (2.54–3.84)*
*p-value*	< 0.001	< 0.001		< 0.001	< 0.001	
**Classmate smoking**						
No one	97 (4.6)	334 (9.2)	Reference	66 (3.4)	197 (5.4)	Reference
Some	254 (15.2)	200 (23.5)	2.85 (2.32–3.49)*	115 (7.3)	111 (13.6)	2.31 (1.83–2.92)*
More than half	245 (29.8)	197 (31.8)	4.99 (4.14–6.02)*	127 (14.9)	135 (21.9)	3.98 (3.17–4.99)*
*p-value*	< 0.001	< 0.001		< 0.001	< 0.001	
**Ever witnessed a teacher smoking in school**						
No	170 (8.0)	279 (10.3)	Reference	91 (4.4)	160 (5.9)	Reference
Yes	397 (19.0)	277 (22.4)	2.27 (1.95–2.64)*	193 (9.2)	194 (16.1)	2.23 (1.77–2.82)*
*p-value*	< 0.001	< 0.001		< 0.001	< 0.001	
**Witness someone smoking inside home in the past 7 days**						
No	260 (8.5)	381 (10.5)	Reference	128 (4.5)	228 (6.2)	Reference
Yes	319 (19.9)	344 (22.9)	2.60 (2.28–2.96)*	177 (10.8)	222 (15.0)	2.64 (2.24–3.11)*
*p-value*	< 0.001	< 0.001		< 0.001	< 0.001	
**Witness someone smoking inside enclosed public places in the past 7 days**						
No	211 (8.6)	382 (10.5)	Reference	98 (3.9)	235 (6.6)	Reference
Yes	389 (17.9)	371 (23.1)	2.32 (2.01–2.67)*	214 (10.3)	217 (13.5)	2.42 (2.08–2.81)*
*p-value*	< 0.001	< 0.001		< 0.001	< 0.001	
**Witness someone smoking at outdoor public places in the past 7 days**						
No	196 (8.7)	335 (10.6)	Reference	99 (4.3)	200 (6.4)	Reference
Yes	410 (17.1)	417 (20.3)	2.16 (1.90–2.46)*	215 (9.4)	248 (12.1)	2.16 (1.82–2.57)*
*p-value*	< 0.001	< 0.001		< 0.001	< 0.001	
**Witness someone smoking at school in the past 30 days**						
No	207 (9.3)	390 (10.8)	Reference	111 (5.1)	240 (6.6)	Reference
Yes	344 (14.6)	317 (21.8)	1.78 (1.52–2.09)*	178 (7.9)	181 (12.8)	1.74 (1.45–2.09)*
*p-value*	< 0.001	< 0.001		0.003	< 0.001	
**Witness someone smoking on TV/in movies in last 30 days**						
No	112 (8.0)	178 (10.3)	Reference	62 (4.7)	104 (6.1)	Reference
Yes	359 (14.3)	348 (14.9)	1.62 (1.38–1.89)*	188 (7.5)	216 (9.3)	1.55 (1.23–1.95)*
*p-value*	< 0.001	< 0.001		0.016	< 0.001	
^1^ Adjusted for age and sex.						

**Table 5:** Anti-tobacco messages and use of tobacco or e-cigarettes among Albanian students in grades 7-10th, by survey year

	Tobacco use			E-cigarettes		
	Survey year		Pooled dataset	Survey year		Pooled dataset
	2015	2020		2015	2020	
	N (%)	N (%)	OR (95% CI)^1^	N (%)	N (%)	OR (95% CI)^1^
**Have seen or heard anti- tobacco messages on social media in last 30 days**						
No	222 (14.1)	351 (14.5)	Reference	114 (7.3)	215 (9.0)	Reference
Yes	361 (11.8)	354 (13.1)	0.88 (0.77-1.00)	177 (6.0)	203 (7.6)	0.87 (0.71–1.07)
*p-value*	0.024	0.145		0.216	0.129	
**Have seen or heard anti- tobacco messages at social events in last 30 days**						
No	200 (14.7)	208 (14.4)	Reference	99 (7.2)	138 (9.9)	Reference
Yes	197 (13.8)	196 (18.4)	1.02 (0.87–1.21)	107 (7.7)	118 (10.9)	1.08 (0.88–1.33)
*p-value*	0.531	0.021		0.572	0.479	
**Have seen signs forbidding buying tobacco in last 30 days**						
No	261 (11.9)	249 (13.4)	Reference	138 (6.2)	162 (8.9)	Reference
Yes	3030 (12.7)	444 (14.0)	1.10 (0.90–1.33)	140 (6.3)	255 (8.0)	0.96 (0.76–1.21)
*p-value*	0.603	0.703		0.960	0.315	

^1^ Adjusted for age and sex

**Table 6:** Advertisements/promotions and use of tobacco or e-cigarettes among Albanian students in grades 7-10th, by survey year

	Tobacco use			E-cigarettes		
	Survey year		Pooled dataset	Survey year		Pooled dataset
	2015	2020		2015	2020	
	N (%)	N (%)	OR (95% CI)^1^	N (%)	N (%)	OR (95% CI)^1^
**Have seen videos on the internet encouraging smoking in last 30 days** ^**2**^						
No	-	369 (11.0)	Reference	-	200 (6.0)	Reference
Yes	-	220 (20.4)	1.94 (1.60–2.36)*	-	147 (13.7)	2.32 (1.82–2.97)*
*p-value*	-	< 0.001		-	< 0.001	
**Have seen tobacco ads at social events in last 30 days** ^**2**^						
No	-	274 (14.0)	Reference	-	162 (8.3)	Reference
Yes	-	179 (25.2)	1.89 (1.42–2.52)*	-	124 (18.5)	2.30 (1.60–3.30)*
*p-value*	-	< 0.001		-	< 0.001	
**Have seen tobacco ads at sales points in last 30 days**						
No	211 (9.6)	287 (11.9)	Reference	162 (8.3)	110 (5.0)	Reference
Yes	229 (17.5)	218 (18.7)	1.75 (1.51-2-02)*	124 (18.5)	127 (10.1)	1.81 (1.42–2.31)*
*p-value*	< 0.001	< 0.001			< 0.001	
**Ever offered free tobacco products from a company**						
No	411 (10.0)	569 (12.3)	Reference	171 (7.2)	209 (5.3)	Reference
Yes	131 (29.2)	114 (36.0)	3.16 (2.62–3.80)*	137 (11.7)	70 (15.1)	2.52 (1.99–3.21)*
*p-value*	< 0.001	< 0.001		< 0.001	< 0.001	

^1^ Adjusted for age and sex. ^2^ Only asked in GYTS 2020

**Table 7:** Knowledge/attitude toward smoking and use of tobacco or e-cigarettes among Albanian students in grades 7-10th, by survey year

	Tobacco use					E-cigarettes			
	Survey year			Pooled dataset		Survey year			Pooled dataset
	2015	2020				2015	2020		
	N (%)	N (%)		OR (95% CI)^1^		N (%)	N (%)		OR (95% CI)^1^
**Awareness of tobacco smoke harm**									
No	199 (27.2)	156 (26.2)		Reference		107 (13.4)	129 (21.9)		Reference
Yes	395 (10.0)	549 (12.5)		0.39 (0.32–0.47)*		203 (5.5)	298 (6.9)		0.39 (0.31–0.50)*
*p-value*	< 0.001	< 0.001				< 0.001	< 0.001		
**Taught about tobacco harms in school during last year**									
No	133 (16.3)	247 (15.2)		Reference		70 (8.9)	140 (8.9)		Reference
Yes	402 (11.3)	358 (12.7)		0.76 (0.62–0.92)*		190 (5.5)	231 (8.1)		0.78 (0.62–0.99)*
*p-value*	0.003	0.106				0.014	0.435		
**Cigarette smoking is joyful**									
No	264 (7.4)	406 (9.8)		Reference		146 (4.3)	238 (5.8)		Reference
Yes	222 (32.0)	215 (34.7)		4.75 (4.03–5.61)*		111 (16.0)	137 (22.1)		3.96 (3.11–5.04)*
*p-value*	< 0.001	< 0.001				< 0.001	< 0.001		
**Willing to use a cigarette if offered by a friend**									
No	324 (8.2)	520 (11.2)		Reference		193 (4.8)	316 (6.9)		Reference
Yes	278 (42.7)	189 (46.7)		6.91 (5.79–8.25)*		116 (19.4)	113 (27.6)		4.26 (3.42–5.31)*
*p-value*	< 0.001	< 0.001				< 0.001	< 0.001		
**It is difficult to quit smoking once started**									
No	238 (18.7)	272 (20.5)		Reference		122 (9.8)	179 (13.8)		Reference
Yes	359 (10.6)	449 (11.9)		0.64 (0.55–0.74)*		183 (5.5)	256 (6.7)		0.61 (0.51–0.73)*
*p-value*	< 0.001	< 0.001				< 0.001	< 0.001		
**Support for smoking bans in indoor public places**									
No	169 (20.8)	213 (22.0)		Reference		89 (10.7)	145 (15.1)		Reference
Yes	386 (10.3)	494 (12.0)		0.51 (0.41–0.60)*		191 (5.4)	277 (6.8)		0.50 (0.40–0.61)*
*p-value*	< 0.001	< 0.001				< 0.001	< 0.001		
**Support tobacco advertising ban**									
No	300 (14.9)	317 (17.10)		Reference		147 (7.3)	185 (10.5)		Reference
Yes	248 (9.6)	373 (11.78)		0.65 (0.55–0.77)*		130 (5.4)	227 (7.0)		0.71 (0.59–0.87)*
*p-value*	< 0.001	< 0.001				0.048	< 0.001		

^1^ Adjusted for age and sex

**Table 8:** Availability of tobacco and use of tobacco among Albanian students in grades 7-10th, by survey year and pooled dataset

	Survey year		Pooled dataset
	2015	2020	
	N (%)	N (%)	OR (95% CI)^1^
**Availability of cigarettes near school**			
No	140 (9.0)	245 (10.7)	Reference
Yes	309 (20.7)	290 (28.3)	2.54 (2.11–3.05)*
*p-value*	< 0.001	< 0.001	
**Refused to be sold cigarettes due to age in last 30 days**			
No	253 (44.5)	134 (44.8)	Reference
Yes	58 (35.0)	101 (47.5)	0.81 (0.56–1.18)
*p-value*	0.106	0.512	
**Difficulty in buying cigarettes**			
Easy	315 (24.7)	196 (36.8)	Reference
Difficult	84 (23.6)	208 (30.1)	0.92 (0.69–1.22)
*p-value*	0.712	0.055	

^1^ Adjusted for age and sex.

## Discussion

The findings of the present study reveal significant temporal changes and trends in tobacco use and e-cigarette consumption among school-going adolescents in grades 7–10 in Albania. The overall increase in tobacco use from 12.86% in 2015 to 14.49% in 2020, and the parallel rise in e-cigarette use, from 6.8% in 2015 to 8.8% in 2020, reflect a shifting landscape of smoking habits among the youth where traditional cigarette smoking is on a decline, but alternative forms of tobacco and e-cigarette use are gaining popularity [30]. The increase in e-cigarette use is consistent with the global trend of significant increases in e-cigarette use among adolescents in various countries, including Georgia, Italy, and Latvia [22]. In our study, cigarette smoking declined from 8.4% in 2015 to 4.3% in 2020 among adolescents which may be due to anti-smoking policies, awareness campaigns, and gradual shifts in social norms as well as the increase in tobacco prices over the years [31]. In Albania, the average price of a pack of cigarettes increased from approximately 200 ALL in 2015 to 250 ALL in 2020, reflecting regular taxation adjustments. Although cigarettes remain relatively affordable for many adolescents, this price increase may have influenced smoking behaviors, particularly among those with limited pocket money. However, further studies are needed to explore changes in smoking intensity among remaining smokers as this decrease may reflect a reduction in the proportion of smokers rather than a decline in the number of cigarettes smoked per use. Meanwhile, the rising consumption of other tobacco products, nearly doubling from 6.35 to 12.25%, and the increase in dual use, points towards a concerning trend of diversification in tobacco use habits among young people. This diversification could be attributed to the introduction of novel tobacco products, evolving social norms, and targeted marketing strategies [32–34]. These findings underscore the need for tailored efforts to prevent the progression of tobacco use and reduce overall prevalence, as well as the importance of addressing specific products and combinations of use among adolescents.

With regards to sociodemographic correlates of tobacco and e-cigarette use, the findings indicated a clear age-related trend, with older students showing notably higher odds of use. Research consistently shows that older students have higher odds of using tobacco and e-cigarettes [35–37]. This trend is particularly pronounced among college students, where e-cigarette use is more likely among those who have used other tobacco products, marijuana, and/or alcohol [36]. However, the prevalence of e-cigarette use is also increasing among younger age groups, with a notable proportion of adolescents and young adults who have never smoked cigarettes trying e-cigarettes [37]. Our findings also indicated gender differences in usage, with females showing significantly lower likelihood of both tobacco and e-cigarette use. For example, while 10.4% of males reported using e-cigarettes in 2020, the prevalence among females was significantly lower at 6.7%. This finding is consistent with other research indicating that tobacco use behaviors often vary by gender, with males typically exhibiting higher rates of usage [38, 39]. The fact that men are more likely than women to smoke is reflected in health statistics, particularly lung cancer, for which smoking is a primary risk factor [40]. These findings underscore the importance of addressing gender-specific influences and behaviors in future public health interventions.

Similar to our study, previous studies consistently show a link between pocket money and substance use among adolescents, with higher amounts of pocket money being associated with an increased likelihood of smoking and using e-cigarettes [41–43]. Affordability of tobacco products is a key factor influencing smoking behavior [43]. As of July 2020, the price of a pack of 20 cigarettes in Albania ranged from 200 ALL (lowest cost brand) to 330 ALL (premium brand), with an average price of 250 ALL (approximately 2.37 USD) [19], of this price, 66.67% was tax consist of 16.67% Value added tax (VAT) and 50% excise tax which is lower compare to other West Balakian countries (Bosnia&Herzegovina, Croatia, Serbia, Montenegro, Macedonia, Kosovo) and far below the WHO benchmark of 70% of the retail price [44]. Despite consistent price increases since 2015, cigarettes remain affordable for a significant portion of the students in this study. This suggests a socioeconomic dimension to smoking behavior, as students with more disposable pocket money have easier access to these products. Research shows a particularly strong association between higher pocket money and substance use among students with better financial resources [42]. Therefore, increasing cigarette prices through higher taxation could be an effective strategy to curb smoking among Albanian youth.

In line with previous studies, our study indicated that parental, sibling, peer/classmate, and teacher smoking can significantly increase the likelihood of tobacco and e-cigarette use among adolescents [45, 46]. This influence is particularly strong when older siblings smoke, and it can persist and even increase over the course of adolescence [46]. Parents who actively discourage smoking can help prevent their children from starting the habit, regardless of their own behavior. This influence is particularly strong in early adolescence, with peers becoming more influential in later adolescence [47]. The findings underscore the importance of family-based interventions and the crucial impact of effective communication and a healthy relationship between parents and children in preventing and reducing adolescent smoking. Witnessing teachers smoking also raises concerns, as their smoking behavior could inadvertently signal approval or normalcy of such habits to impressionable students [48]. To address this concern and strengthen tobacco control efforts in schools, the existing smoke-free policy established by Law No. 9636 in 2007 should be expanded. This could involve implementing stricter regulations that explicitly prohibit the use and possession of all tobacco and nicotine products, including e-cigarettes, on school grounds. This comprehensive ban should encompass not only school buildings but also parking lots, fields, and off-campus school-sponsored events, and should be enforced at all times.

Additionally, environmental exposure, including exposure to smoking at home, in enclosed public places, and through media, has been found to significantly influence adolescents’ attitudes and behaviors toward smoking [49–52]. This exposure can lead to the normalization of smoking as an acceptable behavior, increasing the likelihood of tobacco and e-cigarette use among students [49]. The association between viewing tobacco use in movies and susceptibility to smoking has been well-documented [52], with media exposure to smoking being a key predictor of smoking intentions and behavior in adolescents [50]. Adolescents who read fashion, entertainment, and gossip magazines may be more likely to smoke due to a higher drive for thinness and greater receptivity to cigarette advertisements [51]. While Albania has implemented some tobacco control measures like banning most forms of tobacco advertising since 2001 and 2007 [53, 54], as well as introducing smoke-free policies in public places [54], enforcement and compliance with these laws have been lacking [19, 54].

In addition, this correlation observed between the availability of cigarettes near schools and increased odds of tobacco use reinforced the importance of environmental factors on youth smoking behaviors. Studies have found that higher levels of retail tobacco availability are linked to increased odds of smoking initiation [55], and that the density of tobacco retailers surrounding a school is related to student cigarette access behaviors [56]. Furthermore, the number of tobacco retailers near a school is associated with an increased likelihood of non-smokers being susceptible to future smoking [56]. While Albania has laws setting a minimum age for tobacco sales [54], aimed at limiting youth access, the lack of association between refusal to be sold cigarettes due to age and tobacco smokeing in our study suggests weak enforcement of age verification at retail outlets allowing underage youth to still purchase cigarettes. There are currently no specific laws on strengthening age verification protocols or penalties for non-compliance by retailers. Thus, more field research is necessary to determine whether retailers have implemented robust age verification policies.

The lack of association between anti-tobacco messages and the use of tobacco or e-cigarettes among adolescents in this study. This presents a notable insight into tobacco control efforts in Albania and suggests that the current anti-tobacco messages may not be effectively reaching or influencing the target demographic, or that these messages are not resonating with the youth in a way that deters tobacco and e-cigarette use. On the other hand, the findings highlight the significant impact of advertising and promotions on tobacco and e-cigarette use among Albanian students, underscoring the influential role of marketing in shaping youth behavior [57, 58]. Albania has a ban on most forms of direct and indirect tobacco advertising through the 2006 law, including bans on advertising on television, radio, print media, billboards, and point of sale [59]. Additionally, the law for tobacco ban was amended in 2019 to include e-cigarettes [21]. However, there are no bans on publicizing tobacco industry activities or requiring anti-tobacco advertisements [14]. Additionally, the lack of enforcement and monitoring remains a challenge, and dedicating resources to active enforcement, inspections, and monitoring of tobacco marketing, sales to minors, and smoke-free policies could improve policy effectiveness [54]. These emphasize the need for stricter marketing regulations and more effective anti-tobacco messages to counter the influence of advertising on youth tobacco and e-cigarette use and curb the rise among Albanian students.

In the present study, the observed reduction in tobacco and e-cigarette use among students aware of the harms of tobacco smoke and were taught about tobacco harms at school in the past year highlights the importance of health education in reducing the use of tobacco and e-cigarettes [60]. It has been shown that this education may lead to a significant increase in e-cigarette use, despite the decline in traditional cigarettes, suggesting the need for targeted education [61]. However, our study indicated lower use of e-cigarettes among those who received education about tobacco harms, and this may indicate the sufficiency of the relevant materials and instructions at Albanian schools. On the other hand, attitude-related factors such as the perception of smoking as joyful, perceived as easy to quit, and not supporting the ban on smoking as well as peer pressure were associated with higher odds of tobacco and e-cigarette use, which is supported by previous studies [62, 63]. These attitudes are often influenced by peer pressure and socialization, particularly among adolescents [64]. However, there is also evidence that some smokers use e-cigarettes as a means to quit or reduce smoking [62], suggesting a potential for attitude change and harm reduction. These findings highlight the importance of addressing peer influence and promoting positive attitudes towards tobacco and e-cigarette use among adolescents to mitigate their impact on youth tobacco use.

### Limitation and strengths

To the best of our knowledge, this is the first study to determine the temporal changes and correlates of tobacco use and e-cigarettes among school-going adolescents in Albania. Our study serves as a critical first step in identifying these potential factors by examining individual associations with tobacco use. This approach provides a strong foundation for future research that can build more complex models tailored to program development. However, the findings should be interpreted with consideration of some limitations. Firstly, the study focused on school-going adolescents in Albania and the findings are not representative of those not attending school who may exhibit higher prevalence and distinct risk factors for tobacco and e-cigarette use, and adolescents in other countries and contexts. Secondly, the GYTS relies on self-reported data, which is prone to social desirability and recall biases, despite the survey’s anonymity. This might result in the underreporting of smoking behaviors, especially among adolescents where smoking is socially stigmatized. Thirdly, the cross-sectional design of this study precludes causal inferences; the identified risk factors should be viewed as correlates rather than causes of tobacco and e-cigarette use. Therefore, longitudinal studies are recommended to better understand these causal relationships. Despite these limitations, findings suggest gaps in current anti-tobacco policies in Albania. Amendments to tobacco use laws in 2013, which increased fines for tobacco product advertising, and subsequent amendments in 2019 to regulate e-cigarette use, seem ineffective in reducing adolescent tobacco use. Lastly, our analysis revealed that the prevalence of dual tobacco use is notably lower compared to individual use of tobacco and e-cigarettes. This suggests that the majority of students predominantly use either smoked tobacco or e-cigarettes in isolation. While we have not considered dual tobacco use as the primary outcome variable in this study, we fully acknowledge the importance of investigating factors associated with dual use in future research. The findings highlight the importance of a comprehensive approach that combines fiscal policies like tobacco taxation, stricter enforcement of smoke-free laws, expansion of cessation services, and targeted public education campaigns focused on youth and influencers like parents and peers. Strengthening penalties for violations of tobacco control laws has also shown promise in reducing smoking uptake among youth [59].

## Conclusion

This study reveals a critical shift in smoking behaviors among Albanian adolescents over the study period, with a decline in traditional cigarette use offset by rising e-cigarette and other tobacco product consumption. These trends, driven by sociodemographic and environmental factors such as peer influence, media exposure, and easy access to tobacco near schools, highlight the urgent need for comprehensive interventions. Stricter enforcement of tobacco control laws, increased taxation, and targeted health education are pivotal in addressing these challenges. The findings not only inform Albania’s public health strategies but also provide actionable insights for global efforts to curb adolescent tobacco and nicotine use, emphasizing the importance of adapting policies to emerging trends in youth smoking behaviors.

## Data Availability

The GYTS datasets are publicly available data and are available on WHO NCD Microdata Repository through the following link: https://extranet.who.int/ncdsmicrodata/index.php/catalog/GYTS.
